# Quantitative proteomics of patient fibroblasts reveal biomarkers and diagnostic signatures of mitochondrial disease

**DOI:** 10.1172/jci.insight.178645

**Published:** 2024-10-22

**Authors:** Sandrina P. Correia, Marco F. Moedas, Lucie S. Taylor, Karin Naess, Albert Z. Lim, Robert McFarland, Zuzanna Kazior, Anastasia Rumyantseva, Rolf Wibom, Martin Engvall, Helene Bruhn, Nicole Lesko, Ákos Végvári, Lukas Käll, Matthias Trost, Charlotte L. Alston, Christoph Freyer, Robert W. Taylor, Anna Wedell, Anna Wredenberg

**Affiliations:** 1 Department of Molecular Medicine and Surgery, Karolinska Institutet, Stockholm, Sweden.; 2 Centre for Inherited Metabolic Diseases, Karolinska University Hospital, Stockholm, Sweden.; 3 Department of Medical Biochemistry and Biophysics, Karolinska Institutet, Stockholm, Sweden.; 4 Mitochondrial Research Group, Translational and Clinical Research Institute, Faculty of Medical Sciences, Newcastle University, Newcastle upon Tyne, United Kingdom.; 5 NHS Highly Specialised Service for Rare Mitochondrial Disorders, Newcastle upon Tyne Hospitals NHS Foundation Trust, Newcastle upon Tyne, United Kingdom.; 6 Science for Life Laboratory, School of Engineering Sciences in Chemistry, Biotechnology and Health, KTH-Royal Institute of Technology, Solna, Sweden.; 7 Laboratory for Biomedical Mass Spectrometry, Biosciences Institute, Faculty of Medical Sciences, Newcastle University, Newcastle upon Tyne, United Kingdom.

**Keywords:** Metabolism, Mitochondria, Molecular diagnosis, Proteomics

## Abstract

**BACKGROUND:**

Mitochondrial diseases belong to the group of inborn errors of metabolism (IEM), with a prevalence of 1 in 2,000–5,000 individuals. They are the most common form of IEM, but, despite advances in next-generation sequencing technologies, almost half of the patients are left genetically undiagnosed.

**METHODS:**

We investigated a cohort of 61 patients with defined mitochondrial disease to improve diagnostics, identify biomarkers, and correlate metabolic pathways to specific disease groups. Clinical presentations were structured using human phenotype ontology terms, and mass spectrometry–based proteomics was performed on primary fibroblasts. Additionally, we integrated 6 patients carrying variants of uncertain significance (VUS) to test proteomics as a diagnostic expansion.

**RESULTS:**

Proteomic profiles from patient samples could be classified according to their biochemical and genetic characteristics, with the expression of 5 proteins (GPX4, MORF4L1, MOXD1, MSRA, and TMED9) correlating with the disease cohort, thus acting as putative biomarkers. Pathway analysis showed a deregulation of inflammatory and mitochondrial stress responses. This included the upregulation of glycosphingolipid metabolism and mitochondrial protein import, as well as the downregulation of arachidonic acid metabolism. Furthermore, we could assign pathogenicity to a VUS in *MRPS23* by demonstrating the loss of associated mitochondrial ribosome subunits.

**CONCLUSION:**

We established mass spectrometry–based proteomics on patient fibroblasts as a viable and versatile tool for diagnosing patients with mitochondrial disease.

**FUNDING:**

The NovoNordisk Foundation, Knut and Alice Wallenberg Foundation, Wellcome Centre for Mitochondrial Research, UK Medical Research Council, and the UK NHS Highly Specialised Service for Rare Mitochondrial Disorders of Adults and Children.

## Abstract 2

Quantitative proteomics on fibroblasts from 67 patients with mitochondrial disease and 17 control samples reveals putative bio markers and diagnostic signatures for mitochondrial disorders.

## Introduction

Inborn errors of metabolism (IEM) comprise a group of over 800 disorders marked by the toxic accumulation or depletion of essential metabolites (1, 2). Many of these disorders are monogenic and affect pathways related to carbohydrate, lipid, amino acid, organic acid, peroxisomal, or lysosomal metabolism. Mitochondrial disorders stand out as a heterogeneous subgroup of IEM, where the primary defect interferes with mitochondrial aerobic energy conversion. The clinical presentation of these disorders is diverse, affecting various organs, including the brain, skeletal muscle, heart, and liver — either in isolation or as part of a multisystem phenotype — manifesting at any age (3, 4).

To date, over 400 genes are linked to mitochondrial disease, caused by pathogenic variants in either the nuclear (nDNA) or mitochondrial (mtDNA) genome (5). Many of these variants disrupt the assembly or function of the oxidative phosphorylation (OXPHOS) system, a multicomplex system responsible for synthesizing the vast majority of cellular ATP. Over 90 subunits are organized into 5 complexes comprising a respiratory chain (CI–IV) coupled to an ATP synthase (CV). Most OXPHOS subunits and over 130 proteins required for assembly are encoded on the nuclear genome. An additional 13 essential OXPHOS subunits, 22 tRNAs, and 2 rRNAs, required for mitochondrial translation, are encoded on mtDNA (6).

Conventionally thought only to include variants affecting factors involved in OXPHOS function and assembly, primary mitochondrial disorders now also include a broader spectrum of functions, including mitochondrial morphology and dynamics, coenzyme Q10 biosynthesis, proteases, and a wider field of mitochondrial gene expression (7). Generally, mitochondrial diseases have profound consequences for affected individuals and their families, often resulting in severe, chronic, debilitating diseases that are difficult to manage. The lack of effective treatment options frequently leads to a challenging and uncertain future for those affected.

Historically, most patients were diagnosed by their clinical and biochemical presentations but rarely received a definitive genetic diagnosis. Integrating next-generation sequencing into accredited diagnostic services nearly 15 years ago dramatically improved gene discovery for IEM, but, despite combining clinical, laboratory, and genetic analysis, around 50% of investigated patients remain undiagnosed (8). Often, this can be attributed to a failure to classify variants of uncertain significance (VUS), both in coding and noncoding regions, such as promoter and enhancer regions. Although incorporating transcriptomic analysis into diagnostic pipelines has improved the success rate (9), almost half of patients still fail to receive a definite genetic diagnosis, and further improvements and approaches are required.

Recent advances in mass spectrometry allow for the quantitative detection of large numbers of proteins, potentially adding an additional layer to the diagnosis of patients with IEM. Recent reports integrating multiomics approaches into healthcare have demonstrated the feasibility of this strategy (10–15). Furthermore, besides supporting patient diagnostics by providing instant validation of genomic variants, proteomics also increases our understanding of mitochondrial biology disease mechanisms and can further be used to monitor and validate treatment strategies.

Here, we performed mass spectrometry–based label-free proteomics on primary fibroblasts from a cohort of 67 patients with diagnosed or suspected mitochondrial disease and 17 individuals in a control group to advance diagnostics, identify biomarkers, and gain insights into disease mechanisms. Our data reveal that, despite their heterogeneity, certain clinical symptoms prevail within specific groups of mitochondrial disease. Furthermore, we identify several factors consistently altered in all disease groups, suggesting they could act as potential biomarkers for mitochondrial disease pathology. Importantly, we demonstrate that proteomics can be used to stratify into individual mitochondrial disease groups and even support accurate diagnosis in individual cases.

## Results

### Selection and preparation of the study cohort.

We selected 61 participants diagnosed with mitochondrial disease from the mitochondrial disease databases at the Centre for Inherited Metabolic Diseases (CMMS), Karolinska University Hospital, Sweden or the National Highly Specialized Commissioned Mitochondrial Diagnostic Laboratory, Newcastle upon Tyne Hospitals NHS Foundation Trust, UK. Patients were selected based on the following selection criteria: (a) a known genetic diagnosis, (b) the presence of at least 1 causative gene reported in MitoCarta3.0 (16), and (c) the availability of a primary fibroblast culture. Additionally, 6 participants with suspected mitochondrial disease, carrying VUS, were included. As a control group, we incorporated samples from 17 individuals sourced from an internal database at CMMS who exhibited no signs of mitochondrial disease. Comprehensive details regarding the selection criteria are outlined in the Methods section. The final cohort comprised 84 individuals, including 67 patients with diagnosed or suspected mitochondrial disease due to combinations of pathogenic, likely pathogenic, or VUS variants in 53 different genes (Supplemental Data File 1; supplemental material available online with this article; https://doi.org/10.1172/jci.insight.178645DS1).

**Figure 1:**
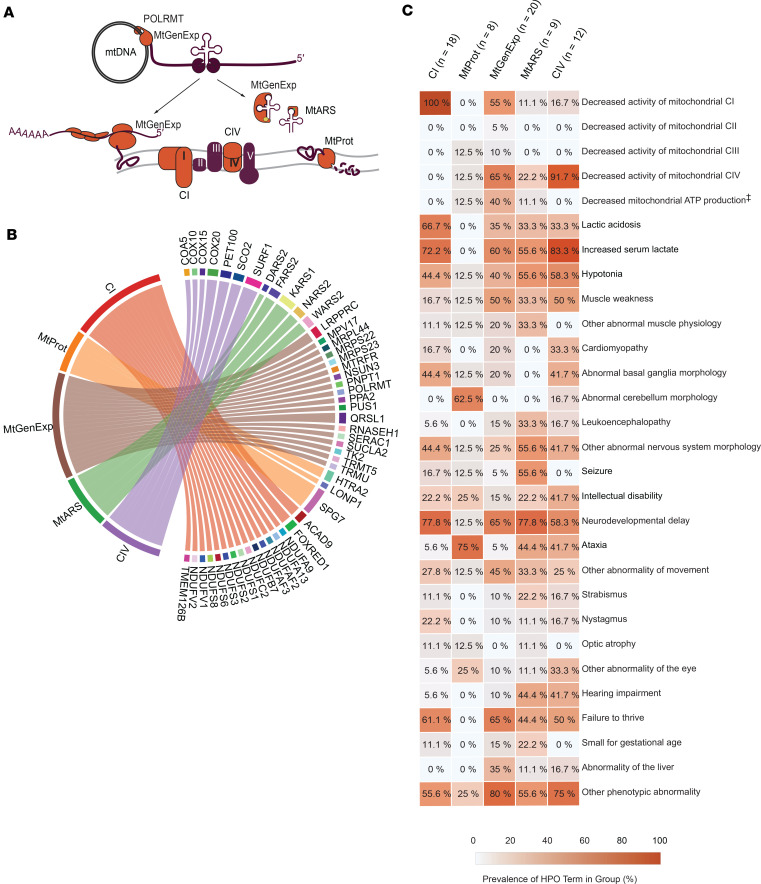
Cohort description and HPO terms in patient groups. Medical chart review revealed HPO terms associated with mitochondrial disease. (**A**) Localization of cohort groups relative to mitochondrial biological function, OXPHOS (CI and CIV), Proteolytic function (MtProt), Aminoacylation of tRNAs (MtARS), mtDNA transcription (MtGenExp), tRNAs modification (MtGenExp) and RNA translation (MtGenExp). (**B**) Chord diagram illustrating cohort genes of interest and their relationship with the groups as defined in the methods and results sections. Line width thickness is equivalent to number of times gene is represented in group. Other genes with variants identified in the cohort but not shown in the plot are *UROD, DPYS, SLC6A17,* and *DHTKD1* (**C**) Prevalence of HPO term in patient group in percentage (%) (further described in Supplemental Data File 1). Number of cases in each group: CI (*n* = 18), MtProt (*n* = 8), MtGenExp (*n* = 20), MtARS (*n* = 9), CIV (*n* = 12) ‡, “Decreased mitochondrial ATP Production” is not an official HPO term.

The patient cohort was further divided into 5 groups based on the biological function of the causative gene or VUS. These groups were defined as: (a) defects in structural components or assembly factors of complex I (CI) (n=18), (b) mitochondrial proteases (MtProt) (n=8), (c) proteins involved in mitochondrial gene expression (MtGenExp) (n=20), (d) mitochondrial aminoacyl transferases (MtARS) (n=9), and (e) structural components or assembly factors of complex IV (CIV) (n=12). This classification enabled us to examine potential mechanistic differences within our study cohort (Figure 1, A and B, and Supplemental Data File 1). Notably, the MtGenExp group is the most heterogeneous, containing genes expressing structural components or assembly factors critical for maintaining, replicating, transcribing, and translating mtDNA.

### Clinical description of the study cohort.

All patients underwent a skin biopsy as part of their clinical and diagnostic investigations, which also involved establishing a fibroblast culture. Of these, 39% of the patients were deceased, 85% of before the age of 10. 79% of the patient group was younger than ten years of age at the time of the biopsy (Table 1), consistent with the previously reported severity of mitochondrial disease (17). A higher proportion of females was observed in the MtARS and CIV groups (Supplemental Data File 1). Medical chart reviews of the patient cohort identified the most common clinical features, which we translated into Human Phenotype Ontology (HPO) terms (Figure 1C and Supplemental Data File 1). HPO term prevalence was determined for each mitochondrial disease group (Figure 1C). As expected, the terms for decreased CI (HP:0011923) and CIV (HP:0008347) activity were frequently found in CI and CIV disease group members, respectively. Interestingly, these terms were also common in the MtGenExp group alongside a decrease in mitochondrial ATP production (not an official HPO term), affirming this group’s general involvement in OXPHOS activity. Other terms, such as increased serum lactate (HP:0002151), hypotonia (HP:0001252), neurodevelopmental delay (HP:0012758), and failure to thrive (HP:0001508) were frequent in most groups (> 40% prevalence), with the notable exception being the MtProt group where these terms were absent or very rarely observed. In the MtARS group, terms such as seizure (HP:0001250) and hearing impairment (HP:0000365) were common, with hearing impairment (HP:0000365) also highly represented in the CIV group. Strikingly, the MtProt group presented with the most unique profile, with terms such as abnormal cerebellum morphology (HP:0001317) and ataxia (HP:0001251) standing out.

### Proteomic studies in fibroblasts.

We applied mass spectrometry–based label-free quantification (LFQ) proteomics on 84 primary fibroblast cultures from our cohort, successfully identifying 5,888 unique proteins with a missing value rate of 37.4% ± 2.8% (mean ± SD) (Supplemental Data File 2). 11% of these proteins (674 out of 5,888) localize to mitochondria, constituting 58% of the mitochondrial proteome (16). Our dataset contained 63% of the known mitochondrial disease genes (5) and covered 61% of proteins included in the CMMS internal IEM database (18) that forms the basis of our diagnostic in silico gene panel (Table 2).

To evaluate the effects of the genetic variants investigated in this study on the corresponding protein levels, we determined the detection rates of these proteins. We identified 34 gene products from the 53 different disease genes (64%) represented in this study, albeit with varying detection rates (Supplemental Figure 1, A and B). To improve data reliability, we filtered out lowly detected proteins, retaining those present in more than 30% of cases in at least 1 group, resulting in a refined dataset of 4,459 proteins, including 536 proteins localized to mitochondria. Missing values were imputed to increase the statistical power of further analysis. Furthermore, the mass spectrometry analysis was performed in 3 separate experiments, requiring a batch correction step to control for technical confounding factors, enabling the quantification of biological variation instead of technical variation (final processed data in Supplemental Data File 3). The bioinformatic processing of data is further detailed in the Methods section.

### Differential expression analysis identifies potential biomarkers of mitochondrial disease.

**Figure 2:**
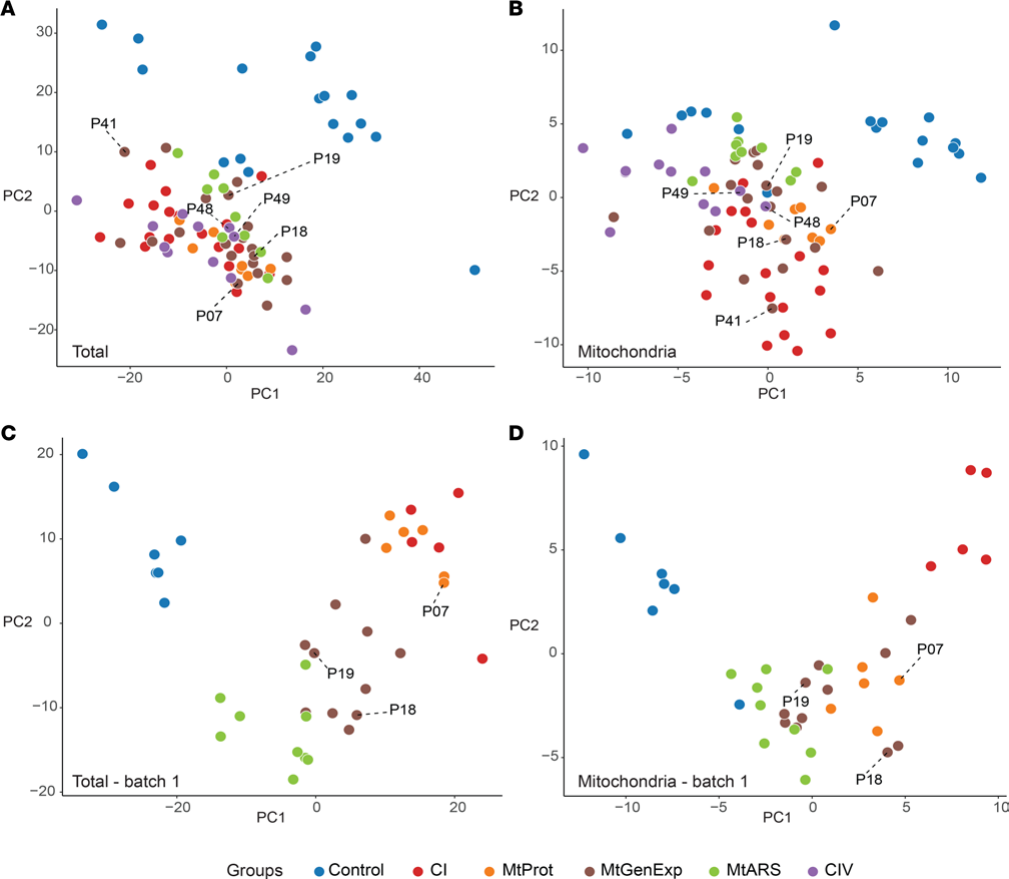
Stratification of mitochondrial disease patients is revealed by proteomics data. Stratification of mitochondrial disease patients in proteomics data. PCA of (**A**) total cell proteomes from the entire patient cohort; (**B**) mitochondrial proteomes from the entire patient cohort; (**C**) total cell proteomes from the patient cohort acquired in the first batch; (**D**) mitochondrial proteomes from the patient cohort acquired in the first batch. Cases with VUS genes are labeled with their respective patient case number (further described in Supplemental Data File 1). VUS cases (genes) include P07 (*HTRA2*), P18 (*POLRMT*), P19 (*QRSL1*), P41 (*MRPS23*), P48 and P49 (*COX20*).

We considered whether untargeted LFQ proteomics could be used to (a) distinguish between control and disease samples, (b) correlate proteomic patterns to specific disease groups, (c) identify putative biomarkers specific to mitochondrial diseases and/or individual disease groups, and (d) improve our understanding of disease mechanisms. Despite applying a batch correction to correct for our experimental set up, a principal component analysis (PCA) of the full proteome (Figure 2A) or mitoproteome (Figure 2B) still revealed a distinct separation between disease samples and controls. Importantly, all VUS cases clustered with the general group of mitochondrial disease. Stratification among different disease groups within the PCA analysis was only achieved in the first batch, presumably due to the exceptionally high data quality, which had a missing value rate of 30.2% ± 1.5% (mean ± SD) (Figure 2, C and D).

**Figure 3:**
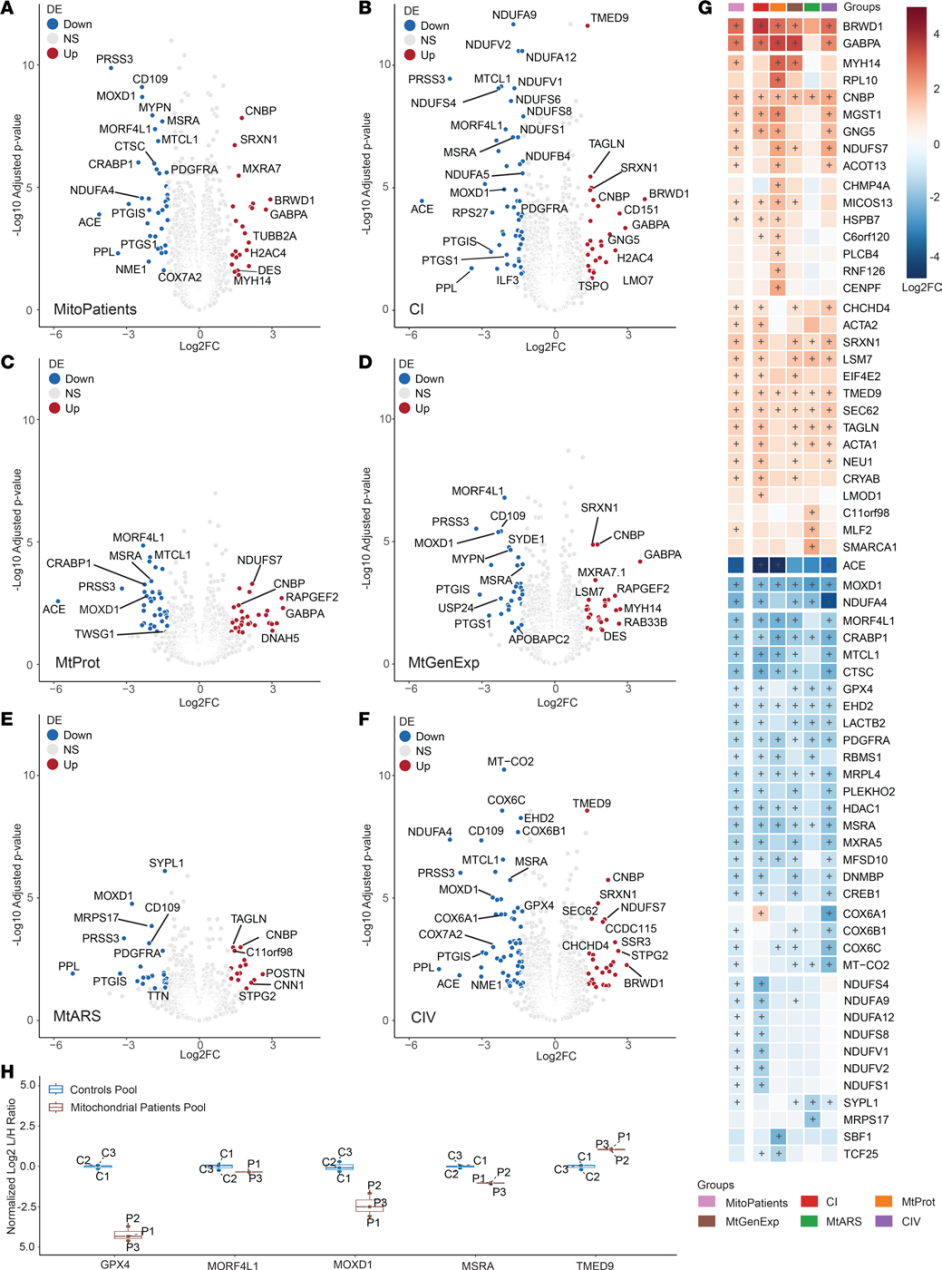
Differential expression analysis reveals biomarkers of mitochondrial disease. Volcano representation of differentially expressed (DE) proteins in groups versus controls in (**A**) Mitochondrial disease patients (MitoPatients); (**B**) CI; (**C**) MtProt; (**D**) MtGenExp; (**E**) MtARS; (**F**) CIV. Significantly changed proteins (Log_2_FC > 1.3 or Log_2_FC < –1.3, adj. *P* < 0.05) are shown in red (increased) and blue (decreased). *P* values were adjusted with Benjamini-Hochberg method and are presented in –Log_10_ scale (–Log_10_ adjusted *P*). (**G**) Heatmap of curated DE proteins, identified in at least 1 of the above comparisons, colored by Log_2_ fold-change versus average control (Log_2_FC) and further annotated with (+) if significantly differentially expressed (adj. *P* < 0.05) in the specific comparison. (**H**) Boxplot of DE proteins of interest, obtained from parallel reaction monitoring mass spectrometry (targeted proteomics) in pooled control (C1–C3) and patient samples (P1–P3). C/P1–C/P3 number indicates technical replicate. Ratios between endogenous peptides (light) and spiked isotope labeled standards (heavy) were determined, normalized to the mean of the control values and Log_2_ transformed (Log_2_ L/H Ratio).

We further applied a linear model for differential expression (DE) analysis using the *limma* package in *R* (further detailed in the methods section), comparing individual patient groups (excluding the VUS cases) as well as the collective mitochondrial disease group (MitoPatients) against the control group. Results were filtered according to their adjusted *P* value (adj. *P* < 0.05) and absolute Log_2_FC > 1.3 (Figure 3, A–F, and Supplemental Data File 4). In total, 178 proteins were significantly changed in at least 1 of the comparisons (Supplemental Figure 2). This protein list was further refined by manual curation, considering technical aspects such as number of peptides identified (removing proteins identified by less than 2 peptides), effects of imputation on DE results (by comparing imputed with nonimputed analysis), susceptibility to batch effects (if there were significant differences in protein intensity between the batches prior to batch-correction), and known biological function. Combining these data quality control steps enabled us to reduce this list to 66 potentially interesting proteins (Figure 3G). Interestingly, 23 proteins were significantly changed in at least 5 of the 6 patient groups compared with the control group. A closer inspection of their data quality (attributed or putative biological function) generated a final list of 7 potential biomarker candidates that were further investigated. For instance, all disease groups presented significantly reduced monooxygenase DBH like 1 (MOXD1) levels and the methionine sulfoxide reductase A (MSRA). In contrast, the transmembrane p24 trafficking protein 9 (TMED9) and the CCHC-type zinc finger nucleic acid binding protein (CNBP) were consistently upregulated. Reduced MOXD1 levels have recently been associated with the activation of ER stress-induced apoptosis (19), while MSRA is involved in the oxidative stress response (20). TMED9 and CNBP have been implicated in autophagy, lysosomal sorting, and the regulation of cytosolic translation, respectively (21, 22). Moreover, the mortality factor 4 like 1 (MORF4L1) and the glutathione peroxidase 4 (GPX4) were frequently downregulated, while the GA binding protein transcription factor subunit α (GABPA) was frequently upregulated (Figure 3G).

To technically validate our findings, we applied a targeted proteomics approach with parallel reaction monitoring (PRM) mass spectrometry using peptide sequences detected in our nontargeted analysis (Supplemental Data File 5). Peptide samples were pooled into common groups, generating a new samples containing either individuals from the control group or patients with mitochondrial disease. These 2 pools were run in triplicate to test for technical variation. This approach confirmed the DE results for 5 of our 7 protein candidates (MOXD1, MSRA, TMED9, MORF4L1 and GPX4) (Figure 3H). The quality of the obtained data was insufficient for GABPA and CNBP, thus, we could not validate the results for these proteins. Further development of a targeted approach for all 7 factors will be needed, and subsequent validation for their use as biomarkers in larger cohorts containing nonmitochondrial diagnosis will be required.

Group-specific changes were observed for CI and CIV, including the downregulation of complex I subunits (NDUFS4, NDUFA12, NDUFS8, and others) in the CI group and a marked decrease of complex IV subunits (COX6A1, COX6B1, COX6C, and MT-CO2) in the CIV group. Remarkably, our analysis also identified factors that exclusively changed in certain disease groups (summarized in Table 3). In contrast, no group-specific changes were observed in the MtGenExp group, probably reflecting the high heterogeneity of this group. Interestingly, the cytochrome c oxidase subunit 6A1 (COX6A1) was downregulated in the CIV but upregulated in the CI defect groups, suggesting that COX6A1 might help distinguish between these 2 mitochondrial disease groups. Overall, our data strongly support the feasibility of stratifying mitochondrial disease groups through proteomic analyses in patient-derived fibroblasts.

### Gene set enrichment analysis reveals pathways altered in mitochondrial disease.

**Figure 4:**
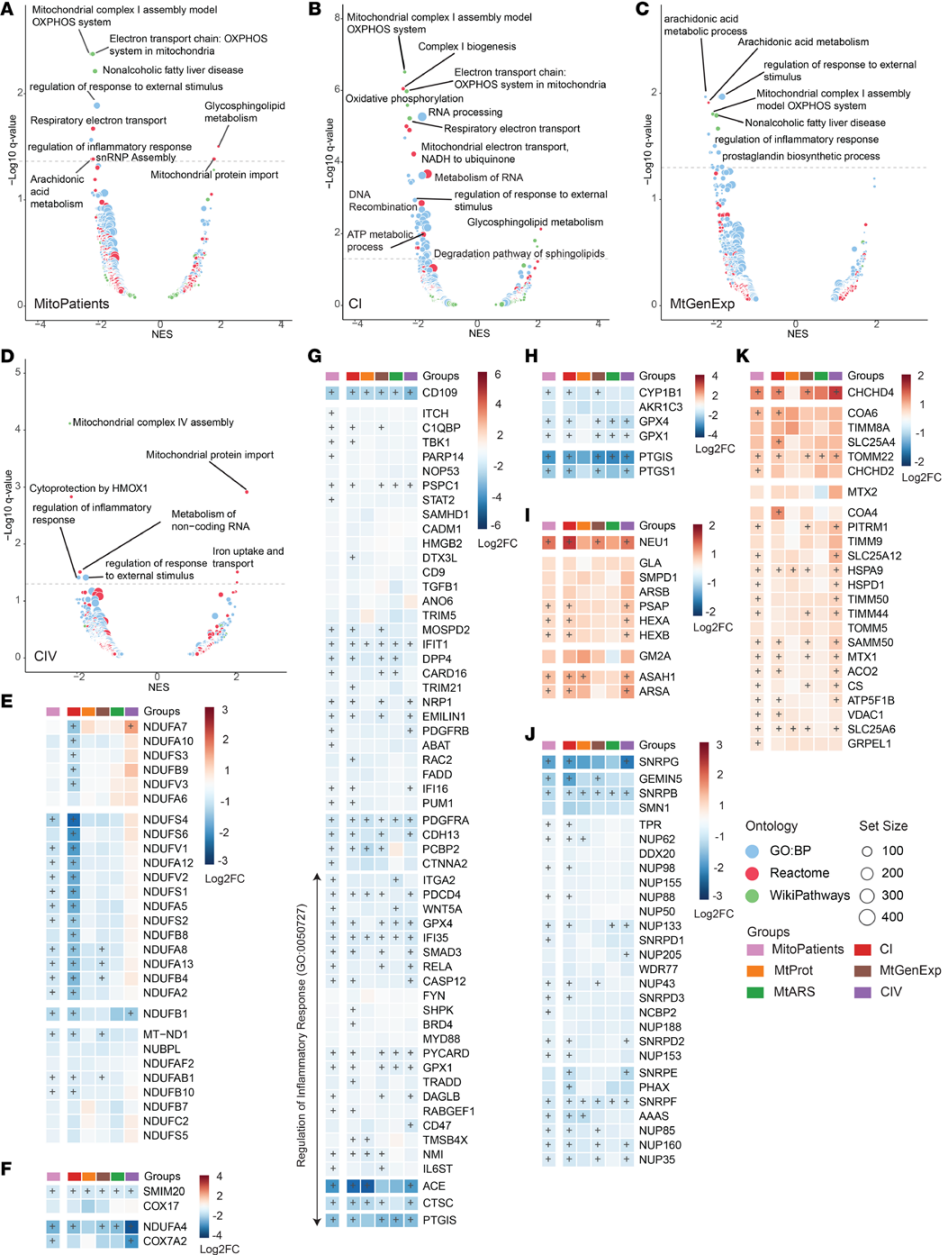
GSEA volcanos and pathway heatmaps. GSEA reveals pathways altered in mitochondrial disease. Volcano plot of GSEA in groups versus controls in (**A**) All Mitochondrial Patients (MitoPatients); (**B**) CI; (**C**) MtGenExp, and (**D**) CIV. Circle diameter denotes set size while color denotes annotation source (blue, GO:BP; red, Reactome; green, WikiPathways). Differentially enriched gene sets are labeled according to a normalized enrichment score (NES) > 1.3 or < –1.3 and a *q* value < 0.05 (presented as –Log_10_ *q* value). Enriched gene sets were further investigated and protein heatmaps were prepared for (**E**) Mitochondrial Complex I Assembly (WP4324); (**F**) Mitochondrial Complex IV Assembly (WP4922); (**G**) Response to External Stimulus (GO:0032101), including Regulation of Inflammatory Response (GO:0050727); (**H**) Arachidonic Acid Metabolism (R-HSA-2142753); (**I**) Glycosphingolipid Metabolism (R-HSA-1660662); (**J**) Metabolism of Noncoding RNA (R-HSA-194441) and (**K**) Mitochondrial Protein Import (R-HSA-1268020). Individual gene set proteins are colored by Log_2_ FC versus average control (Log_2_FC) and further annotated with (+) if significantly differentially expressed (adj. *P* < 0.05) in the specific comparison.

Though individual alterations to the proteomic profile are particularly interesting for the discovery of biomarkers, protein expression changes are relevant in the context of the metabolic pathway involved. Gene set enrichment analysis (GSEA), using consensus pathway annotation catalogues (Figure 4, A–D, and Supplemental Data File 6), further elucidated a general reduction of pathways involved in mitochondrial function (WikiPathways accession no. WP111), inflammation (Gene Ontology accession no. GO:0050727), as well as broader categories such as processes related to immune responses and cellular regeneration (GO:0032101) in the disease cohort (Figure 4A). In contrast, the categories glycosphingolipid metabolism (Reactome accession no. R-HSA-1660662) and mitochondrial protein import (R-HSA-1268020) were overrepresented in the MitoPatient group. Comparisons of individual disease groups to controls only revealed significant changes in the CI, MtGenExp, and CIV groups, with CI representing the most changes (Figure 4, B–D).

Besides categories directly associated with the function of the individual disease groups, such as complex I assembly (WP4324) (Figure 4E) or complex IV assembly (WP4922) (Figure 4F), we noted a significant under-representation of the category regulation of response to external stimulus (GO:0032101) across all disease groups (Figure 4, A–D). This category is strongly influenced by genes involved in inflammation (GO:0050727), with factors such as the angiotensin I converting enzyme (ACE), prostaglandin I2 synthase (PTGIS), and cathepsin C (CTSC) strongly downregulated (Figure 4G). Interestingly, studies in animal models have suggested a potential beneficial effect of ACE inhibitors (ACEI) on mitochondrial function (23–25).

Other significantly changed pathways associated with an inflammatory response include the downregulation of arachidonic acid metabolism (R-HSA-2142753) (Figure 4H) and upregulation of glycosphingolipid metabolism (R-HSA-1660662) (Figure 4I). Arachidonic acid is a precursor of prostaglandins known to act as inflammation mediators (26, 27) that are synthesized by enzymes such as PTGIS and prostaglandin-endoperoxide synthase 1 (PTGS1) (28). This group of metabolites may be potentially interesting biochemical biomarkers and has also been proposed to have deleterious effects on mitochondrial function by activating membrane permeability transition (MPT) and cell death (29, 30). Glycosphingolipids are membrane constituent lipids involved in cell signaling (31), autophagy (32), and enriched in mitochondrial-associated membranes (MAM) (33). We also observed a decrease in the category of noncoding RNA metabolism (R-HSA-194441) (Figure 4J), constituted by proteins involving RNA splicing and subunits of the nuclear pore, thus probably affecting the regulation of mitochondrial function (34) and chromatin modifications (35). Finally, the category of mitochondrial protein import (R-HSA-1268020) was significantly upregulated (Figure 4K), reflective of increased mitochondrial stress (36), which was particularly evident in the CIV group.

Together, these results reveal a general deregulation of proteins related to inflammatory and mitochondrial stress responses in fibroblasts, with both pro- and antiinflammatory factors exhibiting changes in patients with mitochondrial dysfunction. Notably, the data suggest that the molecular origin of a specific, genetically driven mitochondrial dysfunction can be traced based on its proteomic signature. This remains true even if the causative protein itself is undetectable in the analysis, highlighting the potential of proteomics to improve our understanding of mitochondrial dysfunctions.

### Proteomic data support the diagnosis of a mitochondrial disease in an unsolved case.

**Figure 5:**
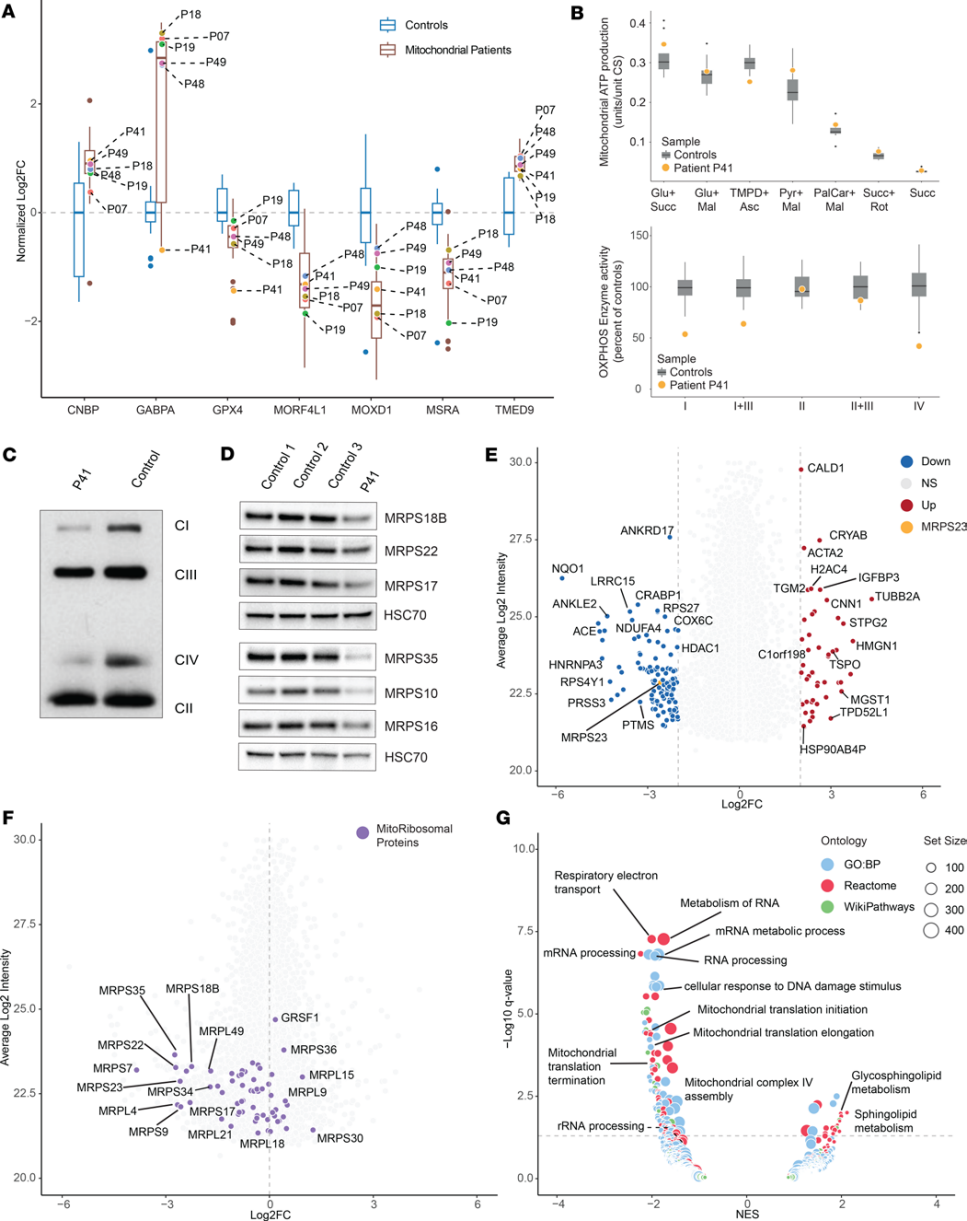
Proteomics analysis supports diagnosis of mitochondrial disease. Proteomics data aids in the diagnosis of an unsolved case. (**A**) Expression levels of the potential biomarkers identified in DE analysis in the 6 VUS cases. Blue boxplots represent the control cohort while brown boxplots indicate the patient cohort. Values are normalized to median intensity of control cohort in Log_2_ scale (Normalized Log_2_FC). Labeled dots denote the VUS cases (genes): P07 (*HTRA2*), P18 (*POLRMT*), P19 (*QRSL1*), P41 (*MRPS23*), and P48 and P49 (*COX20*). (**B**) Mitochondrial ATP production rate (units/unit CS), was determined with the indicated substrate combinations. Boxplots represent the distribution of values for control individuals (*n* = 11, age 12–57 years) with the individual colored circle representing the value determined in the patient. Respiratory chain enzyme activities of complex I, complexes I + III, complex II, complexes II + III, and complex IV were determined in isolated mitochondria and adjusted to CS activity. Results are presented as percentage of mean control values with boxplots representing the distribution of values for control individuals (*n* = 15–42; age 5–70 years) and the individual colored circle representing the value determined in the patient. (**C**) BN-PAGE of mitochondria isolated from muscle from patient (P41) and a control individual. Enzyme complexes I–IV (CI–IV) were detected by Western blot with appropriate antibodies. (**D**) Western blot analysis of patient and control fibroblasts. HSC70 (heat shock protein family A[Hsp70] member 8) was used as loading control and protein signal was determined as described with appropriate antibodies. (**E**) Volcano plot of total proteomes for P41 (*n* = 1) versus controls (*n* = 17). Up or downregulated proteins (Log_2_FC > 2.5 or Log_2_FC < –2.5) are shown in red (increased) and blue (decreased), respectively. MRPS23 protein is labeled in orange. Average Log_2_ Intensities calculated from individual intensities of the entire dataset (patient and controls). (**F**) Volcano plot from **E** with mitochondrial ribosomal proteins emphasized in purple. (**G**) Volcano plot of GSEA analysis of P41 DE results, circle diameter denotes set size while color denotes annotation source (blue, GO:BP; red, Reactome; green, WikiPathways). Differentially enriched gene sets are labeled according to a normalized enrichment score (NES) > 1.3 or < –1.3 and a *q* < 0.05 (presented as –Log_10_ *q* value).

Six of the investigated samples were derived from patients with suspected mitochondrial disease, but a genetic diagnosis had not yet been established. WGS identified VUS in known mitochondrial disease-causing genes in all cases, and the clinical presentations and their laboratory investigations strongly suggested a mitochondrial disease (Supplemental Data File 1). In agreement, evaluation of our initial PCA results (Figure 2A) effectively shows a clustering of these patients with the remaining mitochondrial disease patients. Furthermore, expression levels of the identified putative biomarkers consistently followed the disease cohort with only a few exceptions (Figure 5A). We further analyzed the proteomic profiles of these cases (Figure 5, E–G, and Supplemental Figure 3, A–L), comparing (a) their protein expression with the control groups to identify DE proteins, (b) proteins or pathways related to the function of the VUS gene, and (c) global proteomic changes using GSEA. We identified the most apparent differences in patients P41 (Figure 5, E–G) and P18 (Supplemental Figure 3, D–F). In case P18, we previously identified a homozygous missense mutation in the *POLRMT* gene (c.730C>T) coding for the RNA polymerase mitochondrial (37), promoting a histidine to tyrosine substitution p.(His244Tyr) in NM_005035.3. The patient also carried a VUS in the *SLC6A17* gene [c.335C>T, p.(Pro112Leu)], which encodes for a sodium-dependent amino acid carrier primarily expressed in the brain, and which is associated with intellectual disability (38). Although the candidate protein (POLRMT) was not measured in our sample or controls, we detected several other proteins deregulated in the case (Supplemental Figure 3D). However, the proteomic changes observed in proteins related to the mitochondrial central dogma (Supplemental Figure 3E), as well as the decrease in pathways related to mitochondrial function and translation (Supplemental Figure 3F), are suggestive of a mitochondrial gene expression defect that can be explained by the VUS in *POLRMT*. Further work is required to validate the findings related to this patient, but the proteomic results provide support for a mitochondrial disease due to a mitochondrial gene expression defect. Case P41 was of particular interest, as our genomic investigation identified a homozygous VUS in the mitochondrial ribosomal protein S23, *MRPS23* (c.50G>C), resulting in an arginine to proline substitution p.(Arg17Pro) in NM_016070.3. Additionally, the patient carried variants classified as likely pathogenic (according to ACMG; ref. 39) in dihydropyrimidinase [*DPYS*; c.1010T>C, p.(Leu337Pro)], important in nucleotide metabolism, and uroporphyrinogen decarboxylase [*UROD*; c.1007A>G, p.(Asn336Ser)], which is part of the haem biosynthesis pathway. Although the latter variants are likely to contribute to the clinical presentation of P41, they do not explain the combined mitochondrial CI and CIV deficiency observed in a muscle biopsy from the patient (Figure 5B) as well as the reduced assembly of CI and CIV (Figure 5C). In contrast, MRPS23 is part of the early assembly of the mitoribosomal small subunit (mtSSU) (40, 41), and Western blot analysis revealed reduced steady-state levels of several MRPS subunits (Figure 5D). Moreover, DE analysis displayed significant downregulation of MRPS23 (Figure 5E) and several other MRPSs (Figure 5F), corroborating our Western blot analysis. These results are consistent with a mitochondrial translation defect, as demonstrated by decreased gene sets for mitochondrial translation and electron transport chain (Figure 5G). Finally, WGS did not identify any additional variants in mitochondrial ribosomal proteins associated with disease. Together, our proteomic, genetic, and biochemical evaluation identifies the p.(Arg17Pro) variant in the *MPRS23* gene to be “likely pathogenic” and indicates that it contributes to the clinical presentation in this patient.

## Discussion

Advances in massive parallel DNA sequencing have revolutionized the diagnosis of genetic diseases. Patients with monogenic disorders have significantly benefited with numerous new disease-causing variants being discovered and drastically impacting and improving turnaround times from patient presentation to diagnosis (8, 42–45). Consequently, calls for a personalized approach to patient care are now warranted. For mitochondrial disease, this includes improving the diagnostics and understanding of disease progression. In this regard, skeletal muscle remains the tissue of choice for functional analysis but involves an invasive and logistically complicated approach (46). Here, we used primary fibroblast cultures from 67 patients with diverse known or potential causes of mitochondrial disease to understand their genetic and proteomic profiles. We observed a remarkably responsive proteomic profile, with clear distinctions between patients and controls, among different disease groups and even in individual cases.

We identified 7 potential biomarkers that separated the entire mitochondrial disease group from the controls. Notably, the downregulated MOXD1, MSRA, GPX4, and MORF4L1 are closely associated with cellular stress responses, while the upregulation of GABPA (formerly Nuclear factor erythroid 2-related factor 2 [NRF2]), CNBP, and TMED9 may reflect an effort to counteract a possible redox imbalance in response to a mitochondrial dysfunction (47). Two of the proposed biomarkers (MSRA and GPX4) contain mitochondria-targeted isoforms, and although the incomplete peptide coverage generated by our LFQ approach does not allow us to determine subcellular localization, it is tempting to suggest that these factors primarily respond to the introduced mitochondrial stress. A technical validation using targeted proteomics was pursued for the biomarker candidates, with positive results consistent with the shotgun approach for all except GABPA and CNBP (the latter 2 due to technical inconsistencies). Further validation of these proteins in larger cohorts, other biological materials, and including other pathologies will be required to clarify their potential role as mitochondrial disease biomarkers.

Previously, the fibroblast growth factor 21 (FGF21) and the growth differentiation factor 15 (GDF15) have been used as biomarkers for mitochondrial translation and mtDNA maintenance disorders, or mitochondrial encephalomyopathy, lactic acidosis and stroke-like episodes (MELAS), respectively (48, 49). We detected neither of these growth factors in our dataset, possibly reflecting their extracellular location. Likewise, transcript levels of the transcription factors *ATF4* and *MYC* are increased in several cardiac KO mouse models with mitochondrial dysfunction (50) but were also not observed in our proteomic dataset. Thus, it is likely that a combination of biomarkers should be used to confirm a mitochondrial involvement.

Furthermore, the patient cohort displayed a systemic reduction in members of gene sets related to mitochondrial function and inflammation, while glycosphingolipid metabolism and mitochondrial protein import-related proteins were consistently increased. However, some of these alterations were driven by individual disease groups. For instance, while all groups exhibited an altered inflammatory response, the elevation in glycosphingolipid metabolism and mitochondrial protein import gene sets was predominantly observed in the CI and CIV groups, respectively. Notably, the role of mitochondrial function in inflammatory processes is much debated, and our data also indicate that mitochondrial pathologies can evoke both anti- and proinflammatory responses (51).

The upregulation of several factors involved in proline and one-carbon metabolism was previously reported in the hearts of various mouse models with mitochondrial disease (50). In agreement, we observed similar patterns in our patient cohort, suggesting prominent alterations. However, we did not observe a concurrent downregulation of ubiquinone biosynthesis since these proteins were not detected in our dataset, potentially due to the different tissue types studied. Nonetheless, we previously reported a significant downregulation of ubiquinone steady-state levels in a third of patients with mitochondrial disease in a cohort with 118 patients, suggesting that ubiquinone deficiency is a prominent feature of mitochondrial dysfunction (52).

Previous reports demonstrated the successful implementation of emerging “omics” technologies into diagnostic pipelines of rare diseases (12, 15, 53). The genomic analysis of patient P41 identified 3 possible disease-causative variants, posing challenges in establishing a definitive genetic diagnosis. Variants in *DPYS* and *UROD* have previously been classified as likely pathogenic, while the *MRPS23* variant was reported as a VUS. Genomic variants in *MRPS23* have previously been associated with mitochondrial disease (54), and the data presented here strongly support a mitochondrial diagnosis. Furthermore, the collective loss of multiple MRPs due to pathogenic mutations is well established, serves as a robust diagnostic indicator, and is consistent with the notion that mitochondrial dysfunction also drives disease presentation due to the *MRPS23* variant (14, 55).

Proteomics further highlighted the collective downregulation of CI and CIV assembly categories in their respective disease groups. Historically, such diagnosis has been multidisciplinary, with a combination of clinical phenotyping, morphological and enzyme histochemical investigations, and bioenergetic studies in muscle or skin biopsy material. Targeted genetics and molecular biological methods have then further improved the diagnostic toolbox. With various extensive data acquisition methods being implemented in healthcare, the diagnostic rate can be even further improved. Genomics is already well-established, and untargeted proteomics is the next layer of a multiomics approach to solving mitochondrial disease (46, 56).

In conclusion, our work strengthens the prospect of integrating untargeted proteomics into the routine diagnostic workflow by providing valuable markers of mitochondrial dysfunction, validating genetic findings, and offering insights into the affected cellular processes. Furthermore, the ongoing efforts to develop treatment strategies for mitochondrial diseases require robust, measurable parameters to evaluate new treatment initiatives objectively. Proteomics of patient fibroblasts might provide such therapeutic biomarker parameters (46, 56).

## Methods

### Sex as biological variable.

Both female and male patients were investigated in this study. The proportions of each sex per mitochondrial disease group is further described in Table 1.

### Cohort selection.

To study the potential application of proteomic analysis in the clinical setting, we analyzed a selected cohort of individuals that included cases from the databases at the CMMS, Karolinska University Hospital, Sweden, and the National Highly Specialized Commissioned Mitochondrial Diagnostic Laboratory, Newcastle upon Tyne Hospitals NHS Foundation Trust, UK. The databases contain patients who were referred for investigation of IEM, with a focus on mitochondrial diseases. Routine investigation was performed with a combination of clinical, biochemical and/or genetic analyses to achieve a diagnosis. Biochemical investigation of patients included, among others (52, 57, 58), in-house methods that evaluated mitochondrial ATP production (MAPR) and mitochondrial respiratory chain (MRC) activity in muscle biopsies (59–61). The same tissue was also examined with electron microscopy and histochemical staining (58, 62). Fibroblast cell cultures were established from skin obtained during the muscle biopsies. Genetic analyses included, among others (52, 57, 58, 63), Southern blot for detection of deletions in mtDNA (64), Sanger sequencing of selected genes and mtDNA (65), and more recently, massive parallel whole exome sequencing (WES) (43, 45, 66) or whole genome sequencing (WGS) (8, 42). Inclusion criteria for patients in the study cohort were as follows: (a) having a diagnosis or suspected mitochondrial disease; (b) at least 1 of the causative gene or genes identified as part of the MitoCarta3.0 inventory (16); (c) adequate tissue material (fibroblast cell cultures) was available to be analyzed. Patients with a pathogenic mtDNA variant or mutation in a nuclear gene that could affect mtDNA sequence and copy number were not considered for the study. This exclusion was due to the inherent complexity in analyzing data derived from samples with possible multiple mtDNA deletions and the compounding factor of mtDNA heteroplasmy. One of the study’s goals was to analyze proteomic differences at the group level, and patients whose genetic defects could not be integrated into groups (due to small numbers of equivalent cases) were excluded. The list of patients to be included in the study cohort was further curated with the collaboration of the clinical specialists at both institutions. Control samples were selected from the CMMS internal control database, consisting of individuals (both adults and children) for whom mitochondrial disease was excluded. Control cases were evaluated by clinical specialists at CMMS, who considered results from biochemical, clinical, and genetic evaluations and were deemed not to have mitochondrial disease. These control samples were also selected to better match the age of most of the patient cohort. The patient cohort included 68 patient samples and 17 control samples that, for practical reasons, were run in 3 proteomics batches (Supplemental Data File 1). One subject (P30) was removed from the cohort after failing the quality control criteria of the proteomic data analysis (detailed below in data quality control). The final cohort analyzed, therefore, consisted of 67 patient samples and 17 control samples.

### Sample acquisition and cell culture.

Primary skin fibroblasts derived from biopsies of patient and control participants were cultured in DMEM, high glucose, GlutaMAX Supplement, Pyruvate (Gibco) supplemented with 10% FBS (Gibco) and 1% penicillin/streptomycin under standard conditions. (37°C, 5% CO_2_). Confluent cells were then trypsinized using TrypLE Express Enzyme (1×) (Gibco) and pelleted for further analysis. Briefly, cell media was aspirated from T75 flasks and replaced with 20 mL of warm (37°C) DPBS (Gibco). DPBS was then aspirated, and 3 mL of Trypsin was added to the flasks. Trypsinization was allowed to occur for 5 minutes at 37°C and stopped with 7 mL of cell media. Cells were then pelleted at 600*g* for 5 minutes at room temperature. Media was aspirated and 10 mL of DPBS were used to gently wash and resuspend the cell pellet. Cells were pelleted again at 600*g* for 5 minutes, and the washing procedure was repeated 2 more times for a total of 3 washes. DPBS was completely aspirated after the final wash, and cells were frozen and stored at –80°C.

### Biochemical investigations.

ATP production, respiratory chain enzyme activities, and blue-native electrophoresis (BN-PAGE) were performed as previously described (57, 59–61).

### Protein extraction and Western blot.

Confluent cells were collected with cold PBS by scraping, washed and lysed in RIPA buffer (50 mM Tris-HCl pH 8.0, 150 mM sodium chloride, 1.0% Triton X-100, 0.5% sodium deoxycholate, 0.1% sodium dodecyl sulfate (SDS) supplemented with Complete Protease Inhibitor Cocktail (Roche). Cell debris was cleared by centrifugation, and the protein content of supernatants was determined. Protein suspensions of equal amounts were diluted in RIPA complemented with reducing agent (10× Bolt Sample Reducing Agent, Thermo Fisher Scientific) and loading dye (NuPAGE LDS Sample Buffer, Thermo Fisher Scientific) prior to incubation at 95°C for 5 minutes. Individual proteins were then separated on a 4%–12% precast acrylamide gel (Thermo Fisher Scientific) and transferred to polyvinylidene difluoride (PVDF) membranes with an iBlot Dry Blotting System (Thermo Fisher Scientific). PVDF membranes were blocked with 5% nonfat dried milk in TBS (150 mM NaCl, 50 mM Tris-HCl pH 7.5) containing 0.1% Tween-20 (TBS-T) and incubated overnight at 4°C with primary antibodies diluted in TBS-T with 5% nonfat dried milk. The antibodies used were MRPS18B (Proteintech 16139-1-AP), MRPS22 (Proteintech 10984-1-AP), MRPS17 (Proteintech 18881-1-AP), MRPS35 (Abnova H00060488-B01), MRPS10 (Novus NBP1-83848), MRPS16 (Sigma HPA050081), and the loading control HSC70 (Santa Cruz, sc-7298). The membranes were washed with TBS-T and incubated with an isotype adequate peroxidase-conjugated secondary antibody, anti-rabbit IgG HRP linked (Sigma-Aldrich GENA9340) and anti-mouse IgG HRP linked (Sigma-Aldrich GENA9310). Protein detection was achieved with an ECL Plus Western Blotting Detection System in a Bio-Rad Chemidoc XRS System (Bio-Rad).

### Peptide preparation and liquid chromatography-tandem mass spectrometry.

A LFQ approach in data-dependent acquisition (DDA) mode was applied for total cell proteomics of human fibroblasts. Peptide preparation and liquid chromatography-tandem mass spectrometry were performed with adaptations from previous work (67). Briefly, fibroblast pellets were homogenized with a Teflon-coated pestle in 6 M guanidine hydrochloride and 50 mM Tris-HCl (pH 8.0) for LFQ of the cellular proteome. After sonication and removal of microcellular debris, proteins were reduced with 5 mM 1,4-dithiothreiotol at 55°C for 30 minutes, briefly cooled on ice, and further alkylated in the dark with 2-chloroacetamide for 15 minutes. Protein content was determined with BCA, and 100 μg were digested overnight with 2 μg of Pierce MS-grade trypsin (Thermo Fisher Scientific) at 37°C and gentle shaking. Protein digestion was quenched with 1.2% formic acid (FA), precipitates removed by centrifugation, and supernatant desalted with Empore SPE cartridges (3M) according to manufacturer recommendations. Eluted tryptic peptides were dried in a vacuum concentrator and quantified in 0.5% FA. An aliquot of samples (ca 2 μg) was injected in an UltiMate 3000 nano-UPLC online coupled to a Q Exactive HF hybrid quadrupole-Orbitrap mass spectrometer (Thermo Fisher Scientific). Peptide separation was achieved on a 50 cm long C18 EASY-Spray column (Thermo Fisher Scientific) at 55°C, with the following gradient: 4%–26% of solvent B (98% acetonitrile (ACN) and 0.1% FA) in 90 minutes, 26%–95% of solvent B in 5 minutes, and 95% of solvent B for 5 minutes at a flow rate of 300 nL/minute. Mass spectrometry acquisition was comprised of 1 survey full mass spectrum ranging from a mass/charge ratio (*m/z*) of 350–1,600, acquired with a resolution of R = 120,000 (at *m/z* 200) targeting 5 × 10^6^ ions for a maximum injection time of 100 milliseconds, followed by data-dependent higher-energy collision dissociation (HCD) fragmentations of maximum 18 most intense precursor ions with a charge state 2+ to 7+, using 45 seconds dynamic exclusion. The tandem mass spectra were acquired with a resolution of R = 60,000, targeting 2 × 10^5^ ions for a maximum injection time of 54 milliseconds, setting isolation width to *m/z* 1.4, and normalized collision energy to 33%, setting first mass at *m/z* 100.

### Targeted proteomic analysis.

Biomarker validation was pursued with targeted proteomics in parallel monitoring reaction (PRM). Stable isotope-labeled peptide standards were obtained from Thermo Fisher Scientific (Supplemental Data File 5). Peptides were dissolved in a solution of 20% ACN and 0.1% FA and diluted with 0.1% FA in water to a final concentration of (107.1 fmol/μL). Pooled control and patient samples were prepared by concatenation of equal amounts of peptides prepared from 8 randomly selected control samples and from 9 randomly selected mitochondrial patient samples. Internal standard mixes were then spiked into digested pooled protein samples. Samples were injected in an UltiMate 3000 nano-UPLC coupled to an Orbitrap Eclipse Tribrid mass spectrometer (Thermo Fisher Scientific). Chromatographic separation was achieved on a 25 cm long C18 Aurora Ultimate column (Ion Opticks) at 55°C, applying the following gradient: 4%–26% of solvent B (98% ACN and 0.1% FA) in 60 minutes, 26%–95% of solvent B in 5 minutes, and 95% of solvent B for 5 minutes at a flow rate of 300 nL/minute. Tandem mass spectra were acquired after precursor isolation as defined in an inclusion mass list with 0.7 Th isolation width in the *m/z* range 350–1300 in the quadrupole, at R= 15,000 resolution (at *m/z* 200), targeting 7.5 × 10^4^ ions in a 50 second maximum injection time, with HCD fragmentations at 30% normalized collision energy. Data analysis was performed in Skyline v21.1 (68). Raw data files were imported allowing all isotope labeling types (phenylalanine-[^13^C_9_, ^15^N_1_], leucine-[^13^C_6_, ^15^N_1_], valine-[^13^C_5_, ^15^N_1_] and proline-[^13^C_5_, ^15^N_1_]). Peptide transitions were filtered for *y* ions with 1+, 2+ charge states. Precursor selection was allowing the automatic selection of all matching transitions. Imported data were manually controlled.

### Proteomics data processing.

Raw data were mapped with MaxQuant (v2.2.0.0) (69) against canonical and isoform sequences of the human proteome (UP000005640_9606 from UniProt, accessed in August 2022). Normalization and quantification of identified peptides was achieved in LFQ mode, with FastLFQ deselected, PSM and Protein FDR set at 1%, and match between runs selected. The remaining settings were maintained as defaults. Normalized LFQ intensities were then imported onto Perseus (v.2.0.6.0) (70), where protein groups were further annotated with MitoCarta3.0 (16) and cleaned (removal of “Reverse”, “Potential Contaminant”, and “Only Identified by Site” positive proteins). Samples were assigned to groups in accordance with the information above. Filtering of missing values was performed by removal of protein groups that were not present in 30% of the samples of at least 1 group. Finally, missing values were imputed using the Perseus function “replace missing values from normal distribution” with the parameters width, 0.3; down shift, 1.8; mode, separately for each column.

### Data quality control.

Quality control of the processed data was achieved by analysis of specific high expression nuclear, cytoskeletal, and mitochondrial proteins (Supplemental Figure 4). One sample stood out as significantly different from all the others (P30), with a proteomic pattern that was neither compatible with the clinical description of the case nor with the muscle biopsy result. This sample was reanalyzed (P50) with similar results. WES data revealed no other possible pathogenic variant that would explain the findings. Samples were excluded from the analysis as no new sample could be obtained from the patient. Some cases were run on the proteomics pipeline twice; however, since it was impossible to have technical replicates for all the participants in our study, we decided to remove from subsequent analysis the cases where we had 2 samples. The criteria used to select the samples that were excluded was the missing value rate (Supplemental Figure 5). Samples removed were C5, C19–C29, and P64.

### Data processing.

Processed proteomics data were analyzed in R version 4.4.2 (October 2022) running in RStudio 2023.06.01 (Build 524). Data were generally handled with *tidyverse* (v2.0.0) (71). A linear model for differential expression (DE) analysis was generated in *limma* (v3.57.0) (72) with disease group and technical batch as covariates in the design model. Since sample data were acquired in 3 independent proteomic runs, batch correction using the *removeBatchEffect* function from *limma* was employed for normalization of protein LFQ intensities between sample batches using batch and patient groups as covariates (Supplemental Figure 6, A–D). PCA was performed with *PCAtools* (v2.8.0) (73). Comparisons between patient groups (excluding VUS samples) and controls were performed in *limma* with a moderated *t* test applied for statistical testing with Benjamini-Hochberg correction. GSEA was performed from *limma*-generated log_2_ FC values using *clusterProfiler* (v4.4.4) (74) and *ReactomePA* (v1.40.0) (75) against the Gene Ontology (76), Reactome (77), and WikiPathways (78) consensus annotation databases. Figures were plotted with *ggplot2* (v3.4.2) (79) or with *pheatmap* (v1.0.12) (80) when applicable.

### Statistics.

The patient cohort was characterized according to age at biopsy, status, sex, clinical description in HPO terms, biopsy results, and genetic findings. When applicable, data was presented as mean ± SD. Differences between groups were determined using parametric inferential tests (as described in the data processing section of methods). Depending on test applied, an adjusted *P* value or *q* value under 0.05 was considered significant.

### Study approval.

The study was approved by the Regional Ethical Review Board in Stockholm, Sweden, and the Northeast — Newcastle and North Tyneside 1 Research Ethics Committees. Written informed consent was received prior to participation following the ethical permits.

### Data availability.

The mass spectrometry proteomics data have been deposited to the ProteomeXchange Consortium (81) via the PRIDE partner repository (82) with the dataset identifier PXD047313. Analytical scripts are available upon request. Values for all data points in graphs can be found in the Supplemental Supporting Data Values file.

## Author contributions

The order of cofirst authors was decided alphabetically by last name. A. Wredenberg, A. Wedell, and SPC designed research studies. SPC, MFM, AR, ZK, RW, HB, and LST conducted experiments. SPC, MFM, AR, RW, KN, AZL, NL, RM, ME, and AV acquired data. A. Wredenberg, A. Wedell, SPC, MFM, CLA, LK, MT, CF, RW, and RWT analyzed data. A. Wredenberg, A. Wedell, CLA, and RWT provided reagents. A. Wredenberg, A. Wedell, SPC, MFM, and CF wrote the manuscript. A. Wredenberg, A. Wedell, CLA, RM, and RWT acquired funding for research.

## Supplementary Material

Supplemental data

ICMJE disclosure forms

Supplemental data set 1

Supplemental data set 2

Supplemental data set 3

Supplemental data set 4

Supplemental data set 5

Supplemental data set 6

Supporting data values

## Figures and Tables

**Table 1:**
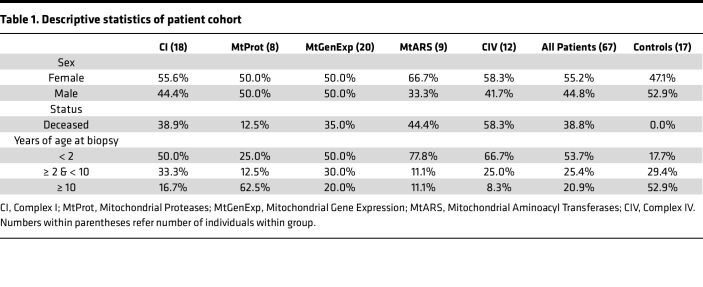
Descriptive statistics of patient cohort

**Table 2:**
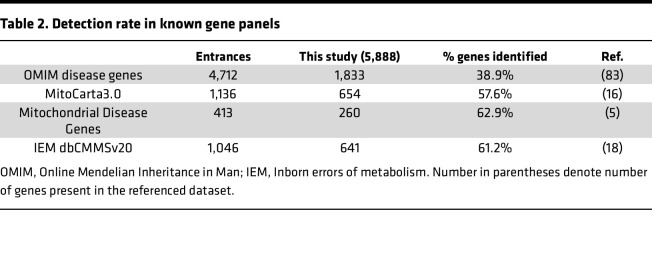
Detection rate in known gene panels

**Table 3:**
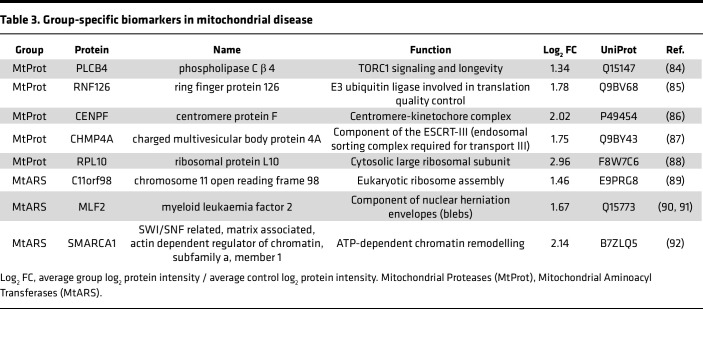
Group-specific biomarkers in mitochondrial disease

## Version 1 09/17/2024

In-Press Preview

## Version 2 10/22/2024

Electronic publication
