# Supplementary material for: Quantitative proteomics of patient fibroblasts reveal biomarkers and diagnostic signatures of mitochondrial disease
Source: JCI Insight. 2024 Oct 22;9(20):e178645. doi: 10.1172/jci.insight.178645 (PMC11530135; doi:10.1172/jci.insight.178645)
Supplement: Supplemental data [file jciinsight-9-178645-s124.pdf]

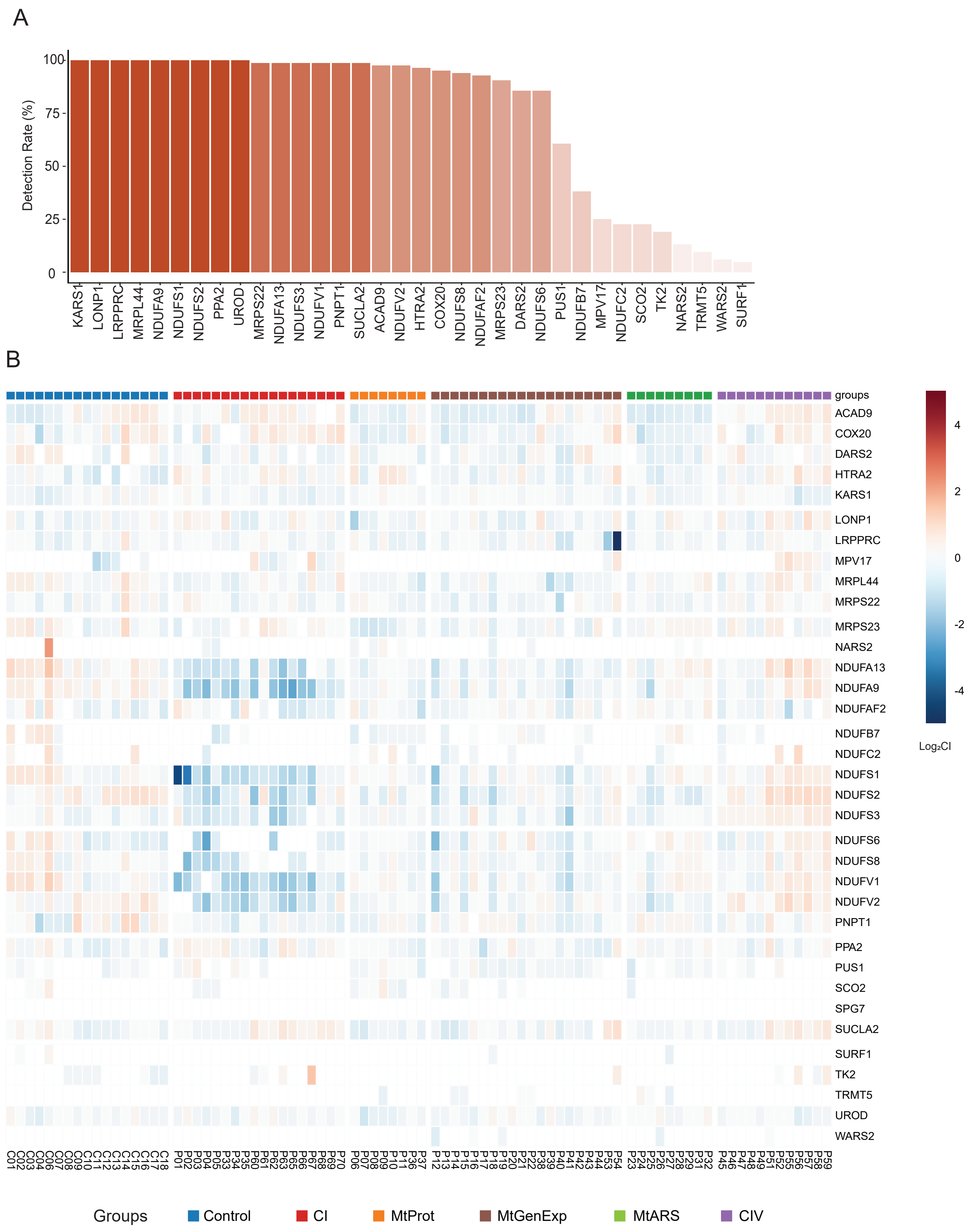

Supplemental Figure 1.
(A) Detection rate (%) of proteins encoded by the genes of interest in the cohort. Rate was determined as number of cases where protein was identified divided by the total number of cases (84). (B) Heatmap showing Log_2 centered intensities (Log_2CI) of proteins encoded by the genes of interest in the patient cohort. Protein intensities were normalized to median intensity value of the entire cohort and Log_2 adjusted (Log_2CI) and are coloured accordingly. Blank cells signify that the protein was not detected in the analysis. Further information in Supplemental Data File 1.

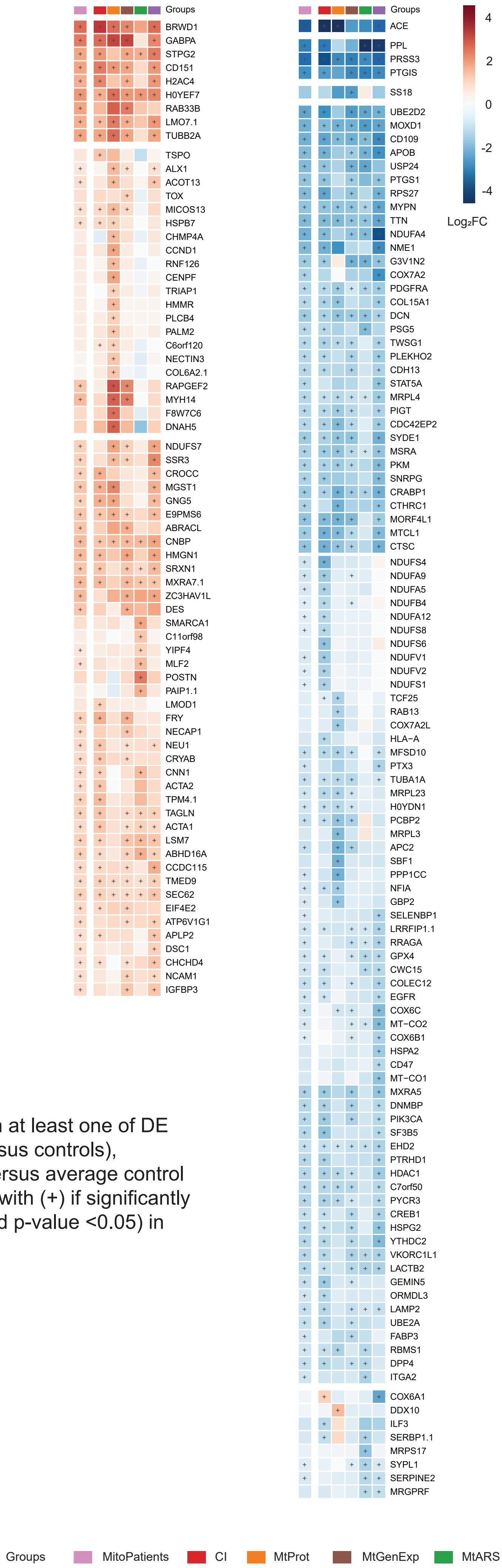

Supplemental Figure 2.
Heatmap of proteins identified in at least one of DE comparisons (cohort groups versus controls), coloured by Log_2 fold-change versus average control (Log_2FC) and further annotated with (+) if significantly differentially expressed (adjusted p-value <0.05) in the specific comparison.

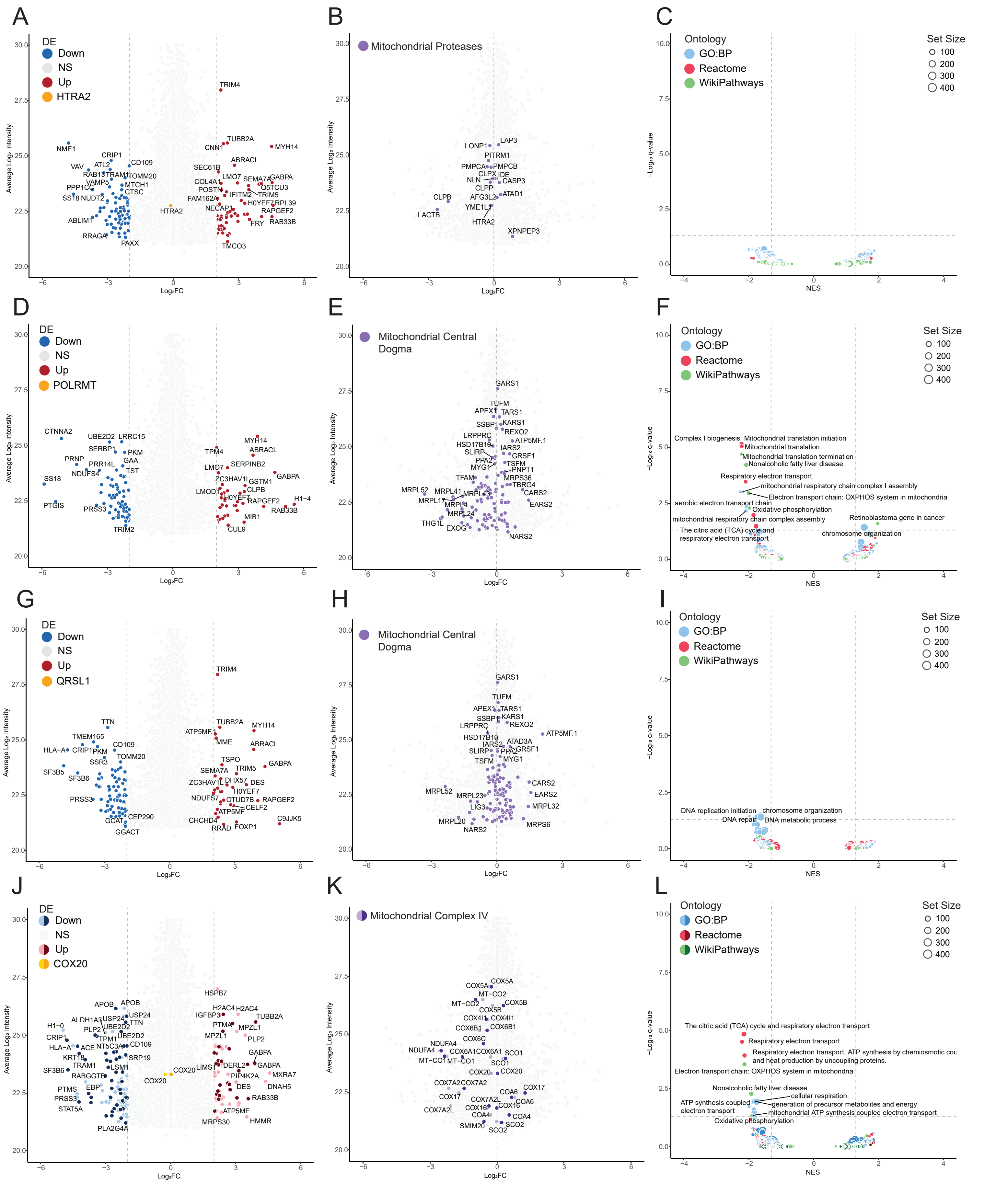

Supplemental Figure 3.
Analysis of VUS cases P07 (variant in *HTRA2*, figures A-C), P18 (variant in *POLRMT*, figures D-F), P19 (variants in *QRSL1*, figures G-I), and P48 and P49 (siblings with variants in *COX20*, figures J-L). COX20 (J-L) patients show in same graph, differently coloured according to case with P48 in a lighter shade, and P49 in a darker shade. (A,D,G,J) Volcano plot of total proteomes for VUS case (n=1) versus controls (n=17). Up- and downregulated proteins ($Log_2FC > 2.5$ or $Log_2FC < -2.5$) are shown in red (increased) and blue (decreased), respectively. If detected, protein corresponding to VUS gene is labelled in orange. Average Log_2 Intensities calculated from individual intensities of the entire dataset (patient and controls). (B,E,H,K) Volcano plots from A/D/G/J with proteins associated with VUS gene (in same pathway or similar function) emphasised in purple (Mitochondrial proteases for B, Mitochondrial central dogma for E and H, Complex IV for K). (C,F,I,L) Volcano plot of GSEA analysis of VUS case DE results, circle diameter denotes set size whilst colour denotes annotation source (blue=GO:BP, red=Reactome, green=WikiPathways). Differentially enriched gene sets are labelled according to a normalized enrichment score (NES) >1.3 or <-1.3 and a q-value <0.05 (presented as $-Log_{10}$ q-value).

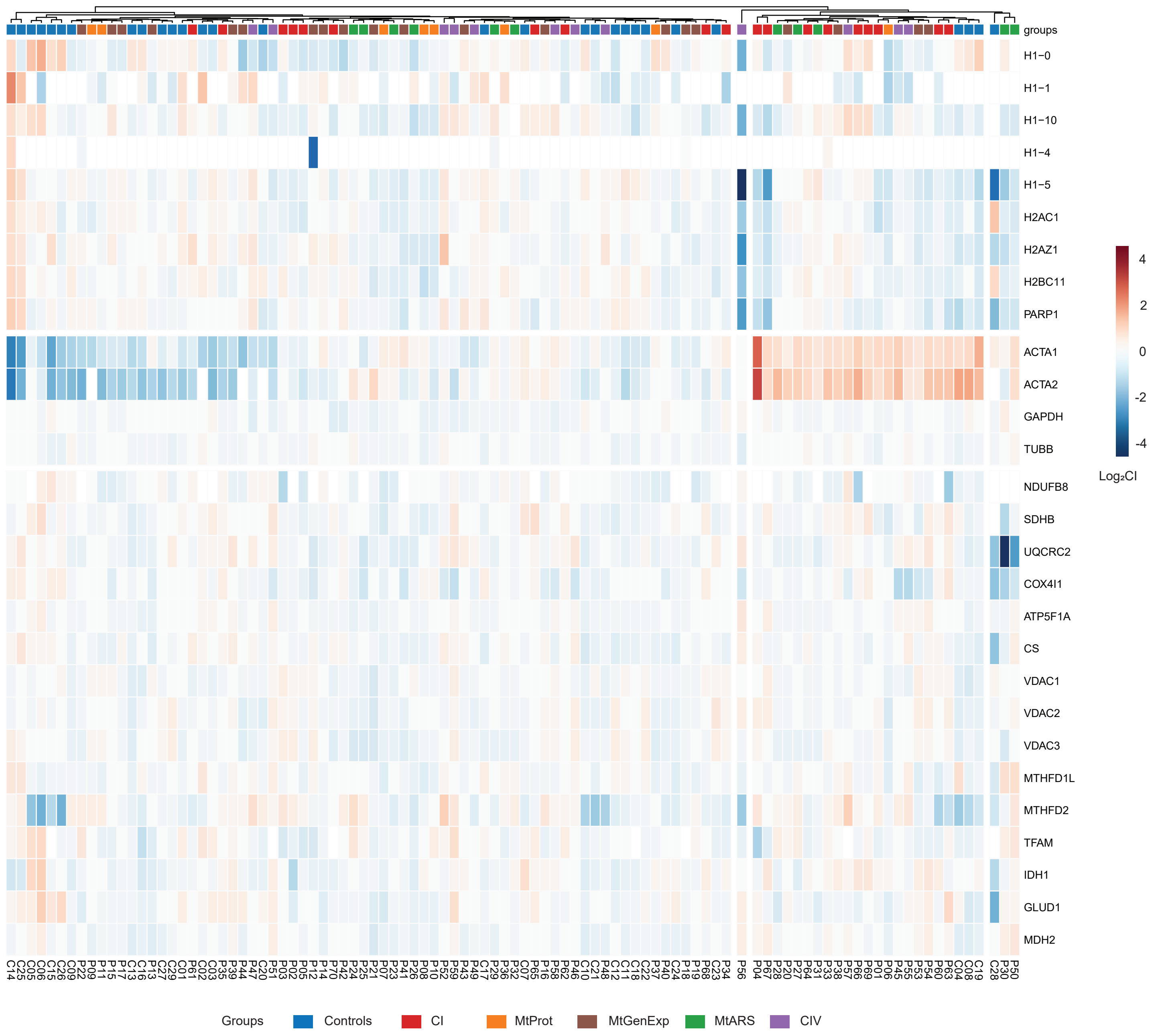

Supplemental Figure 4.

Heatmap of specific high expression proteins involved in nuclear, cytosolic and mitochondrial functions in all samples. Protein intensities were normalized to median intensity value of the entire cohort and Log_2 adjusted (Log_2CI) and are coloured accordingly. Blank cells signify that the protein was not detected in the analysis.

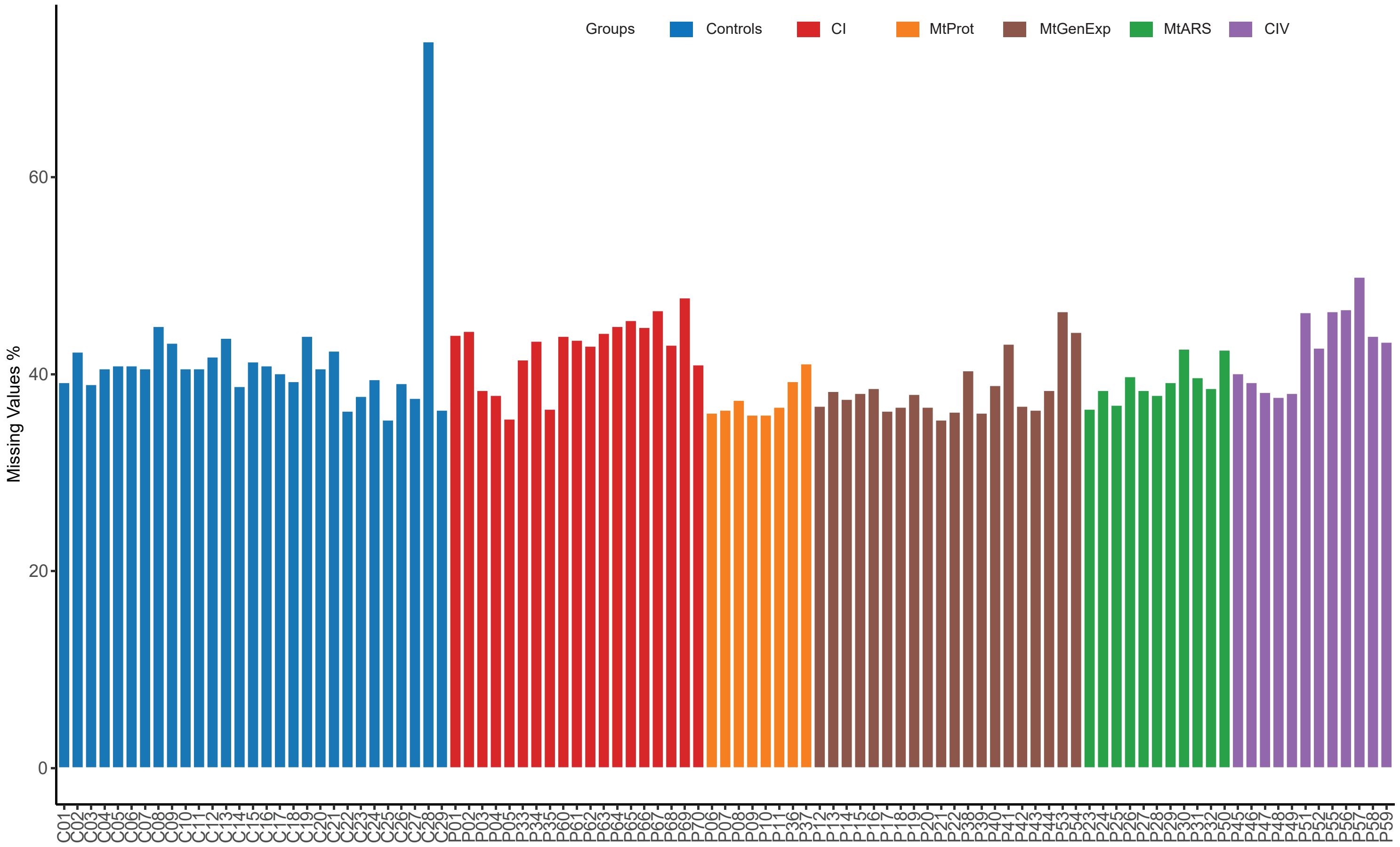

Supplemental Figure 5.
Missing value rate (%) obtained for each sample prior to filtering. Rate was determined as number of missing values divided by total number of proteins detected in the cohort (5888). Colour denotes cohort groups, blue = controls, red = CI, orange = MtProt, brown = MtGenExp, green = MtARS and purple = CIV.

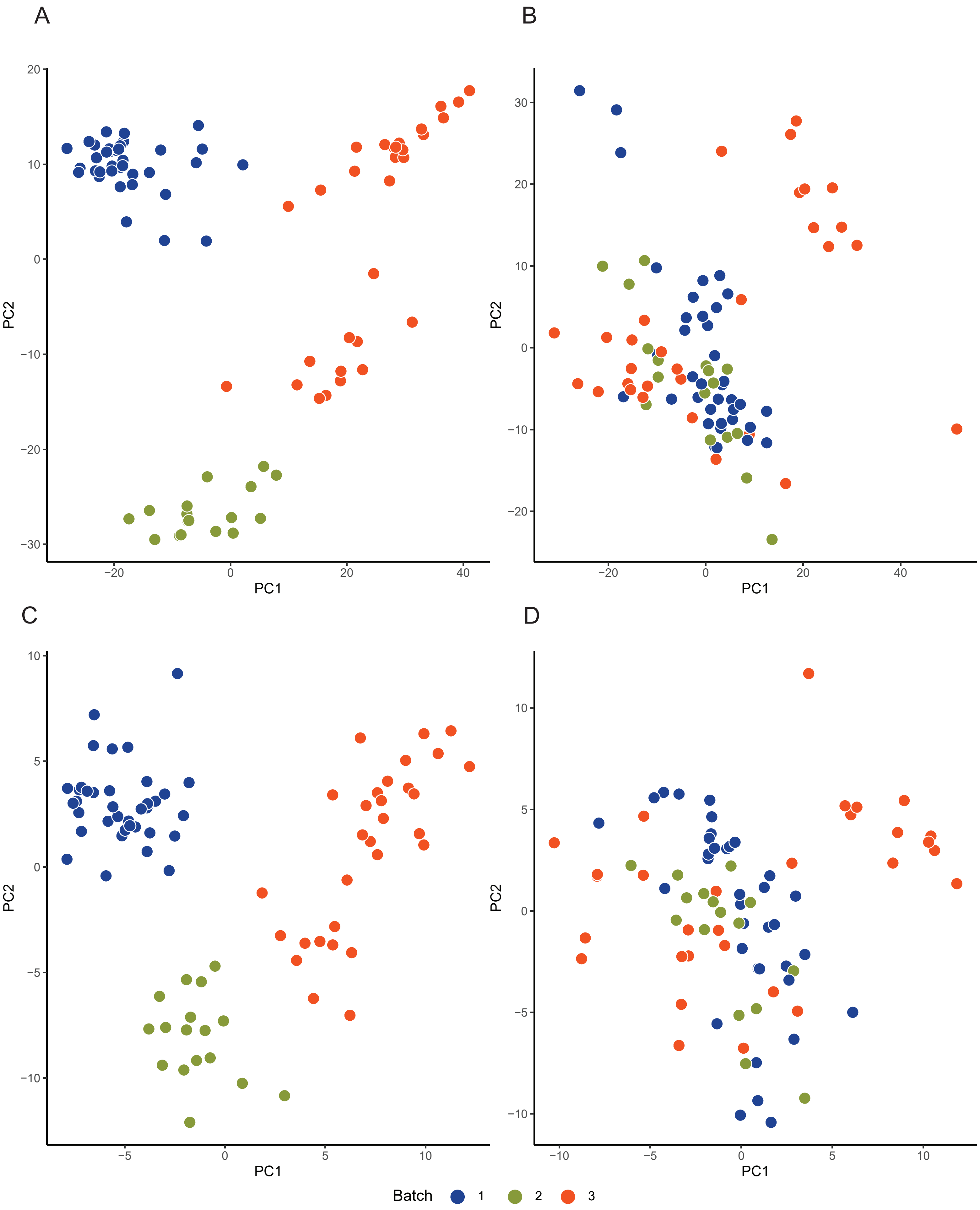

Supplemental Figure 6.
Principal component analysis (PCA) plots before batch-correction of total proteome (A) and mitoproteome (C) datasets. PCA plots after batch-correction of (B) total proteome and (D) mitoproteome datasets. Circle colours denote batch number, blue = batch 1, green = batch 2, orange = batch 3.
